# Synthesis, Characterization, and Electrospinning of a Functionalizable, Polycaprolactone-Based Polyurethane for Soft Tissue Engineering

**DOI:** 10.3390/polym13091527

**Published:** 2021-05-10

**Authors:** Jin-Jia Hu, Chia-Chi Liu, Chih-Hsun Lin, Ho-Yi Tuan-Mu

**Affiliations:** 1 Department of Mechanical Engineering, National Yang Ming Chiao Tung University, Hsinchu 300, Taiwan; 2 Department of Biomedical Engineering, National Cheng Kung University, Tainan 701, Taiwan; kent7210301@gmail.com; 3 Division of Plastic and Reconstructive Surgery, Department of Surgery, Taipei Veterans General Hospital, Taipei 112, Taiwan; chlin12@vghtpe.gov.tw; 4 Department of Surgery, School of Medicine, National Yang Ming Chiao Tung University, Hsinchu 300, Taiwan; 5 Department of Physical Therapy, Tzu Chi University, Hualien 970, Taiwan; hytuanmu@mail.tcu.edu.tw; 6 Department of Sports Medicine Center, Hualien Tzu Chi Hospital, Buddhist Tzu Chi Medical Foundation, Hualien 970, Taiwan

**Keywords:** polyurethanes, two-step reaction, elastomer, functionalization, electrospinning, 2,2-bis(hydroxymethyl)propionic acid

## Abstract

We synthesized a biodegradable, elastomeric, and functionalizable polyurethane (PU) that can be electrospun for use as a scaffold in soft tissue engineering. The PU was synthesized from polycaprolactone diol, hexamethylene diisocyanate, and dimethylolpropionic acid (DMPA) chain extender using two-step polymerization and designated as PU-DMPA. A control PU using 1,4-butanediol (1,4-BDO) as a chain extender was synthesized similarly and designated as PU-BDO. The chemical structure of the two PUs was verified by FT-IR and ^1^H-NMR. The PU-DMPA had a lower molecular weight than the PU-BDO (~16,700 Da vs. ~78,600 Da). The melting enthalpy of the PU-DMPA was greater than that of the PU-BDO. Both the PUs exhibited elastomeric behaviors with a comparable elongation at break ($\lambda =L/L_{0}=$ 13.2). The PU-DMPA had a higher initial modulus (19.8 MPa vs. 8.7 MPa) and a lower linear modulus (0.7 MPa vs. 1.2 MPa) and ultimate strength (9.5 MPa vs. 13.8 MPa) than the PU-BDO. The PU-DMPA had better hydrophilicity than the PU-BDO. Both the PUs displayed no cytotoxicity, although the adhesion of human umbilical artery smooth muscle cells on the PU-DMPA surface was better. Bead free electrospun PU-DMPA membranes with a narrow fiber diameter distribution were successfully fabricated. As a demonstration of its functionalizability, gelatin was conjugated to the electrospun PU-DMPA membrane using carbodiimide chemistry. Moreover, hyaluronic acid was immobilized on the amino-functionalized PU-DMPA. In conclusion, the PU-DMPA has the potential to be used as a scaffold material for soft tissue engineering.

## 1. Introduction

Soft tissue loss caused by trauma, post-tumor resection, aging, etc., can affect aesthetics and even lead to exposure of vital structures of the body [1]. The replacement surgery for treating massive soft tissue loss usually involves the use of autologous tissues, which could cause donor site morbidity [2]. Tissue engineering integrating cells, biodegradable scaffolds, and signaling may provide an alternative to autologous grafts for tissue repair and regeneration [3]. In the process of tissue engineering, the biodegradable scaffold serves as an initial support for cell adhesion and a guideline for microstructural development. Efforts have been made to synthesize polymers that can be used to fabricate scaffolds for repairing or regenerating various tissues. A polymer that is functionalizable and has mechanical properties comparable to the tissue to be replaced is more desired.

Thermoplastic polyesters such as poly(glycolic acid) (PGA), poly(lactic acid) (PLA), polycaprolactone (PCL) and their copolymers have been widely used for tissue engineering applications due to their hydrolytic degradability [4]. The mechanical properties of PGA, PLA, PCL and their copolymers are not always adequate [5,6]. Crosslinked polyesters such as poly(glycerol sebacate) [7] and poly(1,8-octanediol citrate) [8] have several attractive features for use as scaffold materials. It is challenging to fabricate fibrous scaffolds from these crosslinked polyesters owing to their thermoset nature and insolubility [9,10,11]. Most polyesters tend to be less hydrophilic, which is unfavorable for cell adhesion [12]. In addition, chemical modification of the aforementioned polymers is not straightforward.

Polyurethanes are a class of polymers composed of soft and hard segments joined by urethane links [13]. The soft segments are formed from mainly nonpolar polyols, whereas the hard segments from polar diisocyanates and chain extenders. The physiochemical and mechanical properties of polyurethanes are tunable by changing the composition of soft and hard segments. A variety of polyurethanes have been developed for repairing or regenerating soft tissues such as intervertebral discs, blood vessels and articular cartilages [14].

Scaffold materials may need certain functionality to meet their specific application requirements. For example, polymeric scaffolds to be used in contact with blood need to have proper blood compatibility, whereas hydrophilicity and antibacterial capability are important for those to be used as a wound dressing. Specific functionality can be provided if the polymer contains active functional groups such as carboxyl groups or amino groups. For example, Gong et al. prepared a polycarbonate-based polyurethane using dimethylolpropionic acid (DMPA), which contains a carboxyl group, as a chain extender. The carboxyl group-containing polyurethane was then modified by hyaluronan for better blood compatibility [15]. Notably, DMPA, either mixed with polyols or used as a chain extender, has been used to synthesize polyurethanes with tunable hydrophilicity [16], controllable degradability [17], reduced thrombogenicity [18], or stimuli sensitivity [19].

Polyurethanes have been fabricated into porous scaffolds by a variety of techniques [20,21,22]. Among them, electrospinning has received much attention as it requires a relatively simple setup and produces nonwoven meshes containing micro- and nanoscale fibers similar to many extracellular matrix components [23]. Aligned fibers that are capable of guiding the orientation of attached cells can potentially be used to control the neotissue microstructure [24]. The fiber formation during electrospinning is well explained by a theoretical model developed by Reneker and Yarin [25]. Electrospinning has been used in many other biomedical applications [26,27], as well as in fighting against the recent COVID-19 pandemic [28].

In this study, we aimed to develop a biodegradable, elastomeric, and functionalizable polyurethane for soft tissue engineering. PCL was selected as soft segments because of its hydrolyzability and low glass transition temperature (*T*_g_). DMPA was used as a chain extender. Specifically, isocyanate-terminated PCL prepolymer was prepared in the first step and reacted with an equimolar amount of DMPA in the second step to form the polyurethane—that is, DMPA is the sole compound acting as the chain extender. A control polyurethane using 1,4-butanediol (1,4-BDO) as a chain extender was synthesized similarly under the same conditions. The chemical structure of the polyurethanes was confirmed by FTIR and ^1^H-NMR. Their mechanical tensile properties, hydrophilicity, and cytocompatibility were evaluated and compared. The polyurethane using DMPA as a chain extender was further electrospun to fabricate fibrous scaffolds. As a demonstration of the functionalizable carboxyl groups in the polyurethane, gelatin was incorporated to the scaffold by carbodiimide chemistry. Finally, we investigated the possibility of conjugation of hyaluronan on the surface of the functionalizable polyurethane.

## 2. Materials and Methods

### 2.1. Materials

Polycaprolactone diol (PCL diol; PLACCEL 220, Mw = 2000) was obtained from Daicel (Japan). Hexamethylene diisocyanate (HDI, 98.5%) was obtained from Fluka (Switzerland). 1,4-Butanediol (1,4-BDO, 99%) was obtained from Acros (Morris Plains, NJ, USA). Dimethyl acetamide (DMAc, 99.9%) was obtained from Tedia (Fairfield, OH, USA). Stannous octoate (Sn(Oct)_2_), dimethyl sulfoxide-d_6_, *N*-(3-Dimethylaminopropyl)-*N*′-ethylcarbodiimide hydrochloride (EDC), gelatin from porcine skin, Ponceau S solution, and hyaluronan sodium salt (HA) were obtained from Sigma-Aldrich (St Louis, MO, USA). Dibutylamine was obtained from J.T. Baker (Phillipsburg, NJ, USA). 1,1,1,3,3,3-Hexafluoro-2-propanol (99%) was obtained from Matrix Scientific (Columbia, SC, USA). 2,2-Bis(hydroxymethyl)propionic acid (DMPA), 1,4-diaminobutane (1,4-BDA, 98.5%) and N-hydroxysuccinimide (NHS) were obtained from Alfa Aesar (Lancashire, UK). Tetrahydrofuran (THF) was purchased from Macron (Center Valley, PA, USA). Poly-L-lysine (MW = 12,000 Da) was kindly provided by Prof. Jeng-Shiung Jan. PCL diol was dried at 120 °C under vacuum for 4 h prior to use. 1,4-BDO and HDI were purified by distillation and stored over molecular sieves (4 Å, Sigma-Aldrich). DMAc was dried over molecular sieves for more than two days. The water content of PCL diol, HDI, 1,4-BDO, and DMAc was determined by a Karl Fischer titrator (831 Coulometer, Metrohm, Switzerland) to ensure that the water content is less than 100 ppm. DMPA and all other reagents were used as received. For cell culture, Dulbecco’s modified Eagle’s medium (DMEM), penicillin/streptomycin, and trypsin-EDTA were obtained from Thermo Fisher Scientific (Waltham, MA, USA) and fetal bovine serum was purchased from Hyclone (Logan, UT, USA).

### 2.2. Synthesis of Polyurethanes

**Scheme 1:**
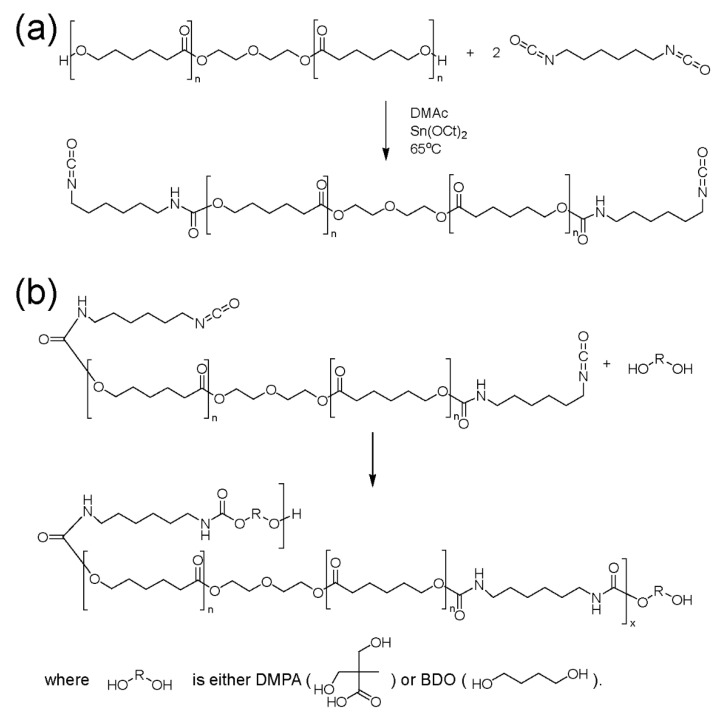
Two-step synthesis of polyurethanes: (**a**) synthesis of isocyanate-terminated prepolymer; (**b**) reaction of the prepolymer with a diol chain extender to establish high-molecular-weight polyurethane.

In this study, polyurethanes were synthesized by two-step solution polymerization (Scheme 1). The synthesis was performed under a dry nitrogen atmosphere in a 250 mL four-necked reactor equipped with a mechanical stirrer. The stoichiometry of the reaction was 2:1:1 of HDI: PCL diol: chain extender (DMPA or 1,4-BDO). In the first step, isocyanate-terminated PCL prepolymer was prepared. A 50% (*w/v*) solution of PCL diol in DMAc was heated to 65 °C in the reactor with continuous stirring. HDI and a catalytic amount of Sn(Oct)_2_ were then added. The prepolymerization reaction was allowed to continue at 65 °C until the isocyanate content reached the theoretical value determined by dibutylamine titration. Subsequently, in the second step, a solution of DMPA or 1,4-BDO was added to the prepolymer solution, initiating chain extension. The reaction was continued until the NCO peak at 2270 cm^−1^ in the Fourier transform infrared (FT-IR) spectrum of the reaction mixture was not detected. The product was precipitated in distilled water, filtered, and dried under vacuum at 60 °C for 24 h. The polymer was designated as PU-DMPA or PU-BDO according to the chain extender used.

### 2.3. Cast Film Preparation

The PU-DMPA and the PU-BDO were dissolved in HFIP and DMF, respectively, to prepare 5% (*w/v*) solutions. The solution was then poured into a Teflon dish (7.5 cm in diameter) and degassed under vacuum at room temperature. Upon the removal of bubbles, the PU-DMPA and the PU-BDO solutions were dried in a fume hood at room temperature and under vacuum at 60 °C, respectively, for 24 h, to form films. The thickness of the film was measured by a custom high-frequency ultrasound system.

### 2.4. Material Characterization

#### 2.4.1. Gel Permeation Chromatography

The molecular weights of the PU-DMPA and the PU-BDO were determined by a gel permeation chromatography and light-scattering (GPC–LS) system (Viscotek, United Kingdom) equipped with two Shodex HFIP columns and three detectors including RI (VE3580, Viscotek), right angle light scattering, and viscometer (Dual 270, Viscotek). HFIP at a flow rate of 1.0 mL/min was used as the eluent.

#### 2.4.2. FT-IR Spectroscopy

The chemical structure of the PU-DMPA and the PU-BDO was examined by infrared spectroscopy. FT-IR spectra of samples were obtained by scanning the samples for 64 times in the wave number range from 600 cm^−1^ to 4000 cm^−1^ at the resolution of 2 cm^−1^ using a Nicolet 6700 FT-IR spectrometer (Thermo Fisher Scientific, Waltham, MA, USA) with an ATR module.

#### 2.4.3. Proton Nuclear Magnetic Resonance

Samples of the PU-DMPA and the PU-BDO were dissolved in dimethyl sulfoxide-d_6_ to prepare 20 mg/mL solutions. ^1^H NMR spectra (500 MHz) were recorded on an Avance 500 NMR spectrometer (Bruker, Germany). Chemical shifts were given in ppm with tetramethylsilane as the standard.

#### 2.4.4. Differential Scanning Calorimetry

The melting behavior and crystallization of the PU-DMPA and the PU-BDO were investigated by a Perkin-Elmer DSC7 (Perkin-Elmer, Norwalk, CT, USA). Samples weighted 6 mg were examined. The sample chamber was purged with nitrogen gas at a flow rate of 50 mL/h. The temperature sequence was arranged as follows: the sample was heated to 280 °C at 10 °C/min, equilibrated at 280 °C, and cooled to −40 °C at 10 °C/min. Then the sample was heated again to 280 °C at 10 °C/min.

#### 2.4.5. Mechanical Tensile Testing

The mechanical behavior of the PU-DMPA and the PU-BDO was estimated by uniaxial mechanical tensile testing. Dog-bone shaped specimens were punched from the PU-DMPA and the PU-BDO cast films using a miniature ASTM D412-C die (gauge length: 16.5 mm; width: 3 mm). The specimen was mounted on an ATS machine (AG-1, Shimadzu, Japan) equipped with a 200 N load cell (Shimadzu, Japan). A tensile test was conducted on under a crosshead speed of 30 mm/min until membrane break. The force (N) and displacement (mm) were recorded. The engineering stress was calculated as force divided by original cross-sectional area and the stretch ratio was calculated as (displacement + 16.5)/16.5. The stress–stretch curves of the specimen were plotted and the ultimate stress, elongation at break and initial modulus of the specimen were obtained.

#### 2.4.6. Water Contact Angle Measurements

The wettability of the PU-DMPA and the PU-BDO cast films was assessed by water contact angle measurements. Water drops of 8 µL were generated by a micropipette and carefully deposited on the surface of the specimens. Photographs of the droplets on the surface were acquired by a digital camera and the contact angles were measured by ImageJ (NIH) with an average of six measurements for each sample.

### 2.5. Cell Adhesion Tests

Human umbilical artery smooth muscle cells (HUASMCs) were used to investigate cell adhesion behavior on the surface of the PU-DMPA and the PU-BDO cast films. HUASMCs were isolated according to an established protocol [9] and cultured in DMEM supplemented by 10% fetal bovine serum and 1% penicillin/streptomycin at 37 °C in a CO_2_ incubator. Before reaching confluence, cells were detached by 0.05% trypsin-EDTA, counted, and resuspended in culture medium for experiments. The PU-DMPA and the PU-BDO cast films were sterilized by immersion in 70% ethanol for 30 min, followed by washing with PBS and then exposure to UV irradiation in a laminar flow hood overnight. The HUASMCs were then seeded on the top of the film or in tissue culture polystyrene dishes at a density of 2.5 × 10^4^/cm^2^. After 24 h, the cell seeded film was washed with PBS and then fixed by 4% paraformaldehyde for 30 min. Images of the attached cells on the film were acquired with an inverted optical microscope (DM IL LED, Leica, Germany) equipped with a digital camera (EOS 7000, Canon, Japan).

### 2.6. Immobilization of Gelatin

**Scheme 2:**
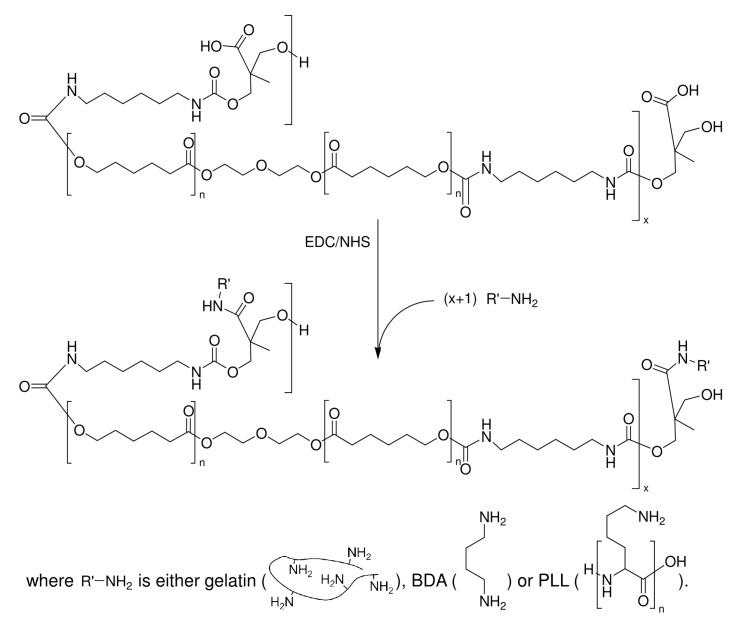
Grafting of primary amines to the PU-DMPA backbone upon carbodiimide-mediated carboxyl activation.

The PU-DMPA cast film (2 cm × 2 cm, ~50 mg) was placed in a solution of EDC (0.2 g) and NHS (0.05 g) in MES buffer (0.5 M, pH = 5.5) (10 mL) for 30 min to activate carboxyl groups on the film surface. After a brief water in MES buffer, the film was transferred to a 10% (*w/v*) gelatin in PBS solution (10 mL) to allow the activated carboxyl groups to react with the amino groups in gelatin for 4 h at 40 °C (Scheme 2). The result of gelatin grafting was verified by FT-IR and Ponceau S staining; the latter was performed following the standard protocol.

### 2.7. Cytotoxicity Tests

The cytotoxicity of the PU-DMPA, gelatin-modified PU-DMPA, and PU-BDO cast films was examined by a direct contact method. HUASMCs were seeded in a 48-well plate at 2.5 × 10^4^ cells per well. Upon cell adhesion, a piece of 2.5 mg of the sterilized PU-DMPA, gelatin-modified PU-DMPA, or PU-BDO cast film was placed on top of the cells. The metabolic activity of the cells was assessed by Alamar Blue assay on days 2, 4, 6 and 8 using the following equation [29]:

$$\mathrm{Percentage}\;\mathrm{of}\;\mathrm{reduced}\;\mathrm{Alamar}\;\mathrm{Blue}\;(\%)=\frac{A_{562}-(A_{620}\times R_{o})}{A_{562}}\times 100$$

where $A_{562}$ and $A_{620}$ are the absorbance of test wells at 562 nm and 620 nm, respectively; $R_{o}$ is the correction factor for filters =$A_{o,562}/A_{o,620}$; $A_{o,562}$ is the absorbance at 562 nm due to oxidized Alamar Blue; and $A_{o,620}$ is the absorbance at 620 nm, due to the same oxidized Alamar Blue. The absorbance was quantified by a microplate reader (Multiskan FC; Waltham, MA, USA, Thermo Scientific). At least five specimens for each group were analyzed.

### 2.8. Electrospinning

PU-DMPA fibrous membranes were prepared by electrospinning using a custom setup which consists of a spinneret (21-gage needle), a syringe pump (KDS-210, KD Scientific, Holliston, MA), a high-voltage DC power supply (AU series, Matsusada Precision, Japan), and a grounded aluminum rotating drum (102 mm in diameter) driven by a high-speed DC motor [9]. Briefly, the PU-DMPA was dissolved in HFIP to prepare 15% (*w/v*) solution. The solution was delivered by the syringe pump at a flow rate of 1.5 mL/h to the spinneret, on which a positive charge of ~10 kV was applied, and electrospun onto the rotating drum. A 25 cm tip-to-drum distance was employed. Fibers were collected for 2 h to form a membrane. The thickness of the membranes, measured by the high-frequency ultrasound system, ranged between 170 and 220 μm. Immobilization of gelatin on the electrospun PU-DMPA membrane was performed following the procedure described earlier. The result was also verified by FT-IR and Ponceau S staining. The porosity of the PU-DMPA electrospun membranes, before and after gelatin immobilization, was measured by a water displacement method [29]. Briefly, a piece of the membranes was immersed in a 5 mL graduated cylinder filled with a known volume of water, $V_{1}$. The increased volume, $V_{2}-V_{1}$, representing the volume of solid material, $V_{solid}$, was measured upon the removal of the air in the pores of the membrane. The volume of the water in the cylinder, $V_{3}$, was measured again after the water-impregnated membrane was removed. The difference between $V_{3}$ and $V_{1}$ was identified as the void volume, $V_{void}$. The porosity was calculated as $\phi =\left(V_{1}-V_{3}\right)/\left(V_{2}-V_{3}\right)$. 

### 2.9. Scanning Electron Microscopy

The morphology of electrospun PU-DMPA fibers before and after gelatin immobilization was examined by scanning electron microscopy (SEM). Pieces of the polyurethane membranes (3 mm × 5 mm) were mounted onto stubs and sputter-coated with platinum (Sputter E-1045, Hitachi, Japan). SEM images were taken by a field emission scanning electron microscope (S-4100, Hitachi, Japan). The voltage used was 15 kV. The vacuum was 5 × 10^−8^ Pa. The diameters of electrospun PU-DMPA fibers, before and after gelatin immobilization, were measured using ImageJ with an average of 30 measurements from three SEM images of each polyurethane.

### 2.10. Amino Functionalization and Modification by Hyaluronic Acid

Based on Scheme 2, the PU-DMPA was amino-functionalized by two approaches. In one approach, 1,4-BDA was grafted directly onto the PU-DMPA cast film following the procedure described earlier; the resultant membrane is designated as PU-DMPA-BDA. In the other approach, the PU-DMPA (1 g) was dissolved in THF (50 mL) to prepare a 2% (*w/v*) solution. An excess amount of EDC (0.72 g) and NHS (0.18 g) in methanol (12 mL) was gradually added to the PU-DMPA solution. After reaction for 30 min, a solution of poly-L-lysine (0.06 g) in methanol (1.5 mL) was added to the solution. The reaction continued for 24 h with agitation. The solution was then casted in a Teflon dish and dried at room temperature to obtain a membrane, designated as PU-DMPA-PLL. The result of amino functionalization was verified by FT-IR and Ponceau S staining.

For immobilization of hyaluronic acid (HA) on the amino-functionalized PU-DMPA, EDC and NHS were added to 0.5% (*w/v*) HA in MES buffer; the final concentrations of EDC and NHS were 6% (*w/v*) and 1.5% (*w/v*), respectively. The amino-functionalized PU-DMPA film (2 cm × 2 cm, ~50 mg), either PU-DMPA-BDA or PU-DMPA-PLL, were added to the HA buffer (10 mL) with stirring at 40 °C for 24 h.

### 2.11. Platelet Adhesion Testing

Human whole blood was collected from healthy human donors. Anticoagulated blood (blood/anticoagulant ratio of 9:1 (*v/v*)) was centrifuged (Model 2420, Kubota, Japan) at 1500 rpm for 15 min to obtain plasma. The PU-DMPA, PU-DMPA-BDA, PU-DMPA-PLL, PU-DMPA-BDA-HA and PU-DMPA-PLL-HA membranes with a size of 1 cm × 1 cm were incubated in the plasma at 37 °C for 1 h. The membranes were then washed with PBS to remove unattached platelets and fixed with a 2.5% glutaraldehyde solution at 4 °C for 2 h. Upon drying through graded alcohols, the membranes were sputter-coated with platinum and the morphology of platelet on the surfaces of the membranes was examined by scanning electron microscopy.

### 2.12. Statistical Analysis

All the data are presented as mean ± standard deviation. The Student’s *t* test was used to compare the mechanical properties and the contact angles of the PU-DMPA and the PU-BDO. One-way ANOVA in conjunction with Holm-Sodal post hoc procedure was performed to examine the difference in cytotoxicity. The level of significance was set at 0.05. A * denotes *p* < 0.05, while ** denotes *p* < 0.001.

## 3. Results and Discussion

The goal of this study is to synthesize an elastomeric polyurethane that can be electrospun and contains carboxyl groups for functionalization. The PU-DMAP and the PU-BDO were synthesized by two-step polymerization under the same conditions. The time required to complete the chain extension of the PU-DMPA and the PU-BDO was 4 days and 24 h, respectively, which indicates the very different reaction activity between DMPA and 1,4-BDO as a chain extender. The molecular weights and molecular weight distributions of the polyurethanes were obtained from GPC results (see Supplementary Figure S1 for GPC chromatograms). The molecular weight of the PU-DMPA (Mw = ~16,700 Da, PDI = 1.31) was significantly lower than that of the PU-BDO (Mw = ~78,600 Da, PDI = 1.60), probably because the hydroxyl groups of DMPA reacted less readily with isocyanate-terminated PCL prepolymer owing to the steric hindrance.

**Figure 1:**
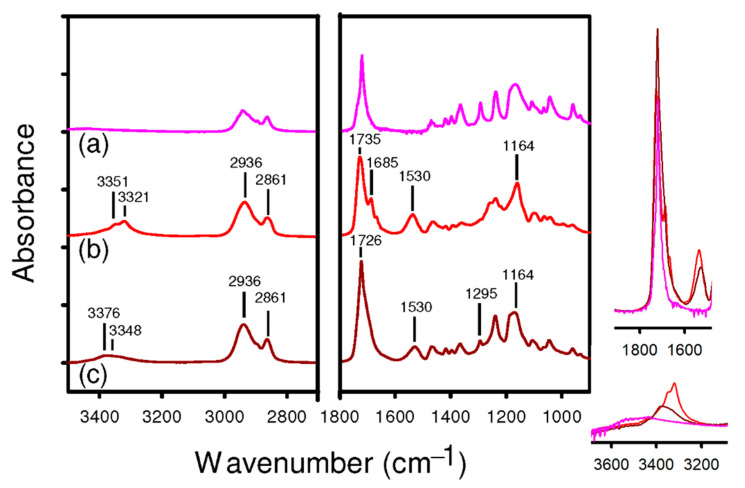
ATR-FTIR spectra of the PCL-diol (**a**), the PU-BDO (**b**) and the PU-DMPA (**c**).

Figure 1 shows the FT-IR spectra of the PU-DMPA and the PU-BDO. The absence of peaks around 2270 cm^−1^, which is a characteristic absorption for N=C stretching, indicates that isocyanates were completely reacted. The emergence of peaks around 1530 cm^−1^, 1700 cm^−1^, and 3300 cm^−1^, which were assigned to the vibrations of N-H bending, C=O stretching, and N-H stretching of the urethane group, respectively, demonstrates the formation of the urethane linkage. 

Generally, C=O stretching vibration of esters and urethanes manifests itself as peaks between 1600 cm^−1^ and 1800 cm^−1^. In the PU-BDO spectrum, the peak at 1735 cm^−1^ and the shoulder peak at 1685 cm^−1^ were assigned to free ester or urethane carbonyl groups and hydrogen-bonded (H-bonded) ester or urethane carbonyl groups, respectively [30,31]. In the PU-DMPA spectrum, although no separate peaks were observed in this region, the peak at 1726 cm^−1^ was strong and broad compared to the two peaks for the PU-BDO. Note that, in addition to the carboxyl group in soft segments and urethane groups in hard segments, there are carboxyl groups in the hard segments and all these groups might contribute to H-bonded carbonyl groups. Both the PU-BDO and the PU-DMPA exhibited 2936 cm^−1^ and 2861 cm^−1^ absorptions, corresponding to symmetric and asymmetric C-H stretching from CH_2_, respectively. 

The peaks around 3300 cm^−1^ were assigned to N-H stretching of urethane amide (for both the PU-DMPA and the PU-BDO) or O-H stretching in the carboxyl group (for the PU-DMPA only). The shifting of the peaks to the low wave number for both the PU-DMPA and the PU-BDO also indicates the formation of hydrogen bonding.

**Figure 2:**
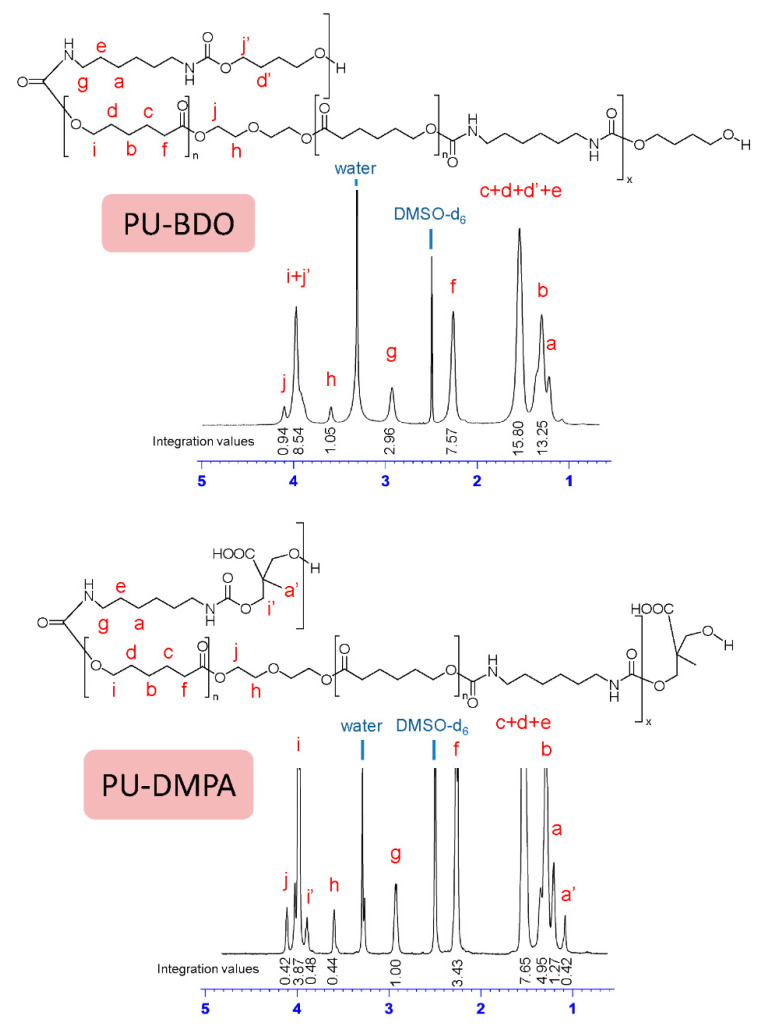
^1^H-NMR of the PU-BDO (**top**) and the PU-DMPA (**bottom**).

Figure 2 shows the ^1^H-NMR spectra of the PU-DMPA and the PU-BDO with each peak assigned. The PU-DMPA and the PU-BDO spectra shared many common peaks, including: 1.2 ppm (peak a), 1.3 ppm (peak b), 1.5 ppm (peaks c-e), 2.25 ppm (peak f), 2.95 ppm (peak g), 3.6 ppm (peak h), 3.98 ppm (peak i), and 4.12 ppm (peak j). These peaks were assigned to proton signals of HDI and PCL units. Peaks d’ and j’ in the PU-BDO spectrum were assigned to proton signals of 1,4-BDO units, whereas peaks a’ and i’ in the PU-DMPA spectrum were DMPA, indicating the chain extenders were incorporated into the polymers, respectively. The results of ^1^H-NMR were consistent with the findings in the FT-IR; both support that both the polyurethanes were synthesized as designed.

**Figure 3:**
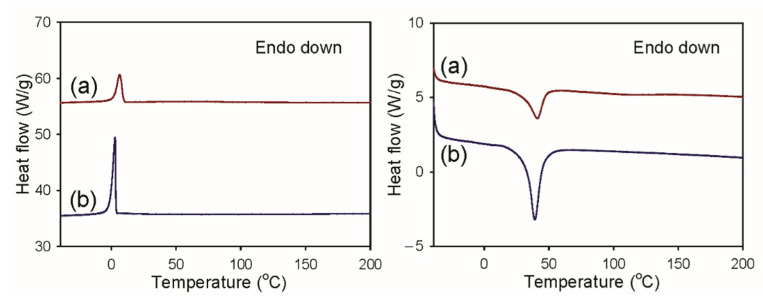
DSC crystallization curves (**left**) and second melting curves (**right**) of the PU-BDO (**a**), and the PU-DMPA (**b**).

Figure 3 shows DSC curves of the PU-DMPA and the PU-BDO. The glass transition temperature (*T*_g_) of both the polyurethanes were below room temperature, which indicates that both the polyurethanes are in a rubbery state at room temperature or under physiological conditions. The PU-DMPA had a slightly lower *T*_g_ (2.5 °C) compared to the PU-BDO (6.8 °C). Both the PU-DMPA and the PU-BDO displayed melting peaks, which is due to the crystallinity of PCL segments. Similarly, the melting temperature (*T*_m_) of the PU-DMPA (39.2 °C) was slightly lower than that of the PU-BDO (41.3 °C). The reduction in *T*_g_ and *T*_m_ might be attributed to the compromised crystallinity of PCL soft segments caused by hydrogen bonds between the hard segments (N-H in urethane linkage and additional O-H owing to the carboxyl group in DMPA) and the soft segments (PCL carbonyl groups) [32]. Additionally, the asymmetric side chains of DMPA in hard segments, including a methyl group and a carboxyl group, might impede an ordered packing of hard segments, which can act as a physical crosslinker. Interestingly, the melting enthalpy of the PU-DMPA was significantly greater than that of the PU-BDO, which might be explained by the additional hydrogen bonding introduced by the carboxyl groups of the PU-DMPA.

**Figure 4:**
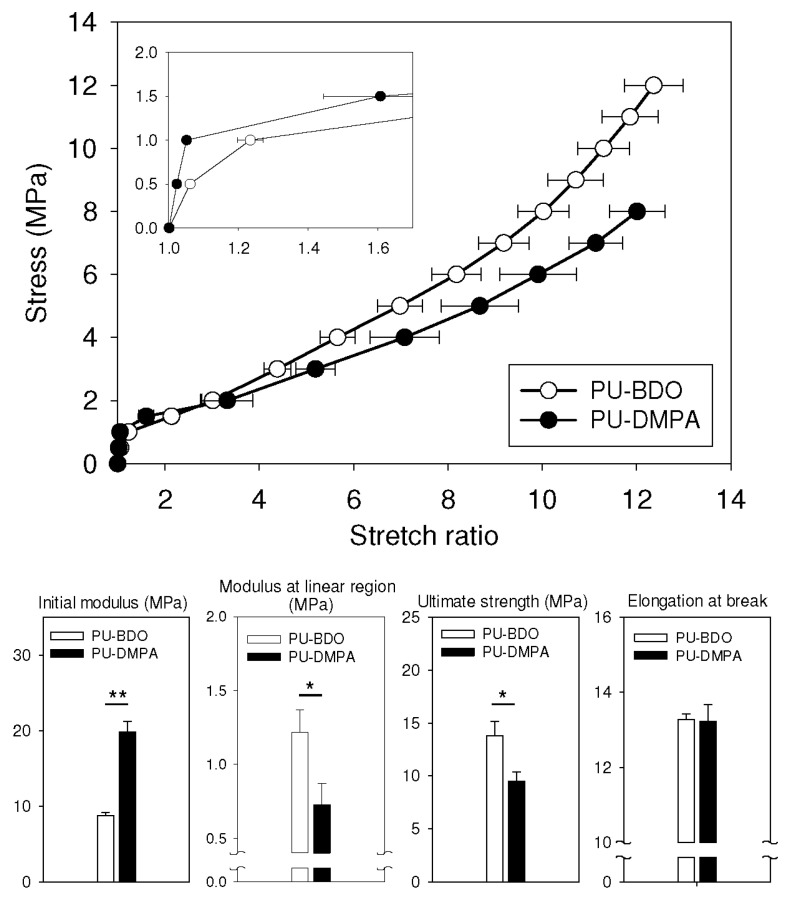
Average stress–stretch curves and mechanical properties of the PU-DMPA and the PU-BDO. * denotes *p* < 0.05, while ** denotes *p* < 0.001.

Figure 4 shows the average stress–stretch curves of the PU-DMPA and the PU-BDO and the comparisons of their mechanical properties. Both the polyurethanes exhibited elastomeric behavior with a comparable elongation at break ($\lambda =L/L_{0}=$13.2). The PU-DMPA had a higher initial modulus (19.8 MPa vs. 8.7 MPa) and a lower linear modulus (0.7 MPa vs. 1.2 MPa) and ultimate strength (9.5 MPa vs. 13.8 MPa) than the PU-BDO. Note that the mechanical properties of the PU-BDO are comparable to those reported by Hong et al. [18]. The elasticity of polyurethanes is derived from the phase separation of the soft and hard segments in a way that the hard segment domains serve as crosslinks among the relatively amorphous soft segment domains. The side chains of DMPA, in contrast to the linear 1,4-BDO, might impede the packing of hard segments of the PU-DMPA and hence contribute to the phase mixing between soft and hard segments, leading to a compromised linear modulus and ultimate strength [33]. The greater initial modulus of the PU-DMPA, which was estimated before the stretch ratio reached 1.2, might be attributed to the additional hydrogen bonding introduced by the carboxyl groups of the PU-DMPA. Recently, Mamidi et al. fabricated carbon nano-onions reinforced PCL composite nanofibers for pH-responsive drug release [34]. The incorporation of carbon nano-onions enhanced the tensile properties of PCL nanofibers. When compared with their composite nanofibers, the PU-DMPA cast film was stronger and more flexible, probably owing to the characteristic interactions between soft and hard segments. The modulus of the PU-DMPA was comparable to some biological soft tissues, such as skin [35] and blood vessels [36], and it is thus particularly suitable to be used in these areas. 

**Figure 5:**
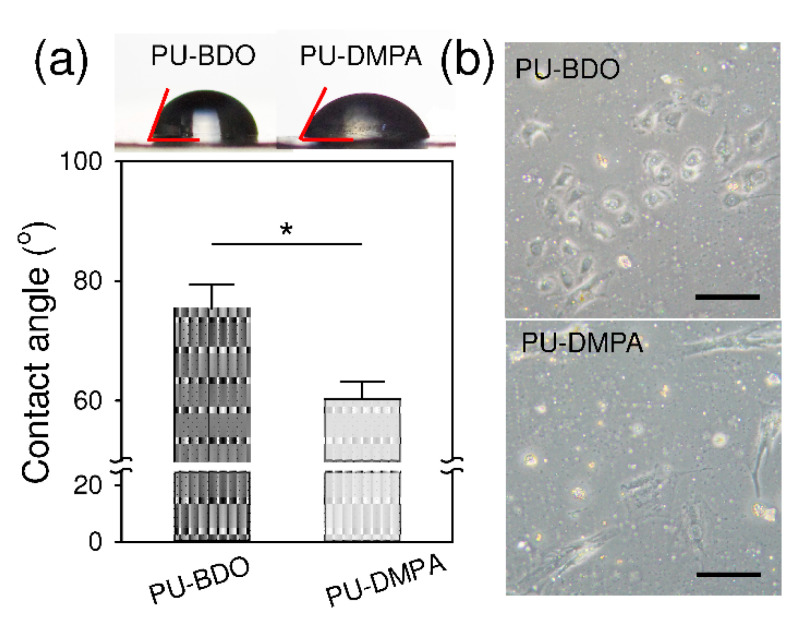
(**a**) Representative photographs of water droplets on the surface of the PU-BDO and the PU-DMPA cast films and the comparison of their contact angles. (**b**) Morphology of HUASMCs adhered on the surface of the PU-BDO and the PU-DMPA cast films after one day of culture; scale bar = 100 µm. * denotes *p* < 0.05.

Scaffold materials for tissue engineering should have proper surface hydrophilicity for cells to adhere to. A moderate surface wettability (50–70°) has been shown to favor cell adhesion and growth [37]. Figure 5a shows that the water contact angle of the PU-DMPA (~60°) was significantly smaller than that of the PU-BDO (~75°) and in the range that supports cell adhesion [37]. The improved wettability might be due to the presence of carboxyl groups in the PU-DMPA backbone, implying that the carboxyl group of DMPA remains intact upon reaction. The result is consistent with Chen et al. [16]. Figure 5b shows the morphology of HUASMCs on the surface of the PU-DMPA and the PU-BDO cast films. The cells adhered and spread on the PU-DMPA surface. Particularly, most of the cells displayed lamellipodia, which suggests that they were migrating. The morphology of the cells on the PU-BDO surface was quite different: most of the cells were round in shape and only a few spread out—that is, the PU-DMPA had better cell adhesion than the PU-BDO, which might be due to the better hydrophilicity of the PU-DMPA.

**Figure 6:**
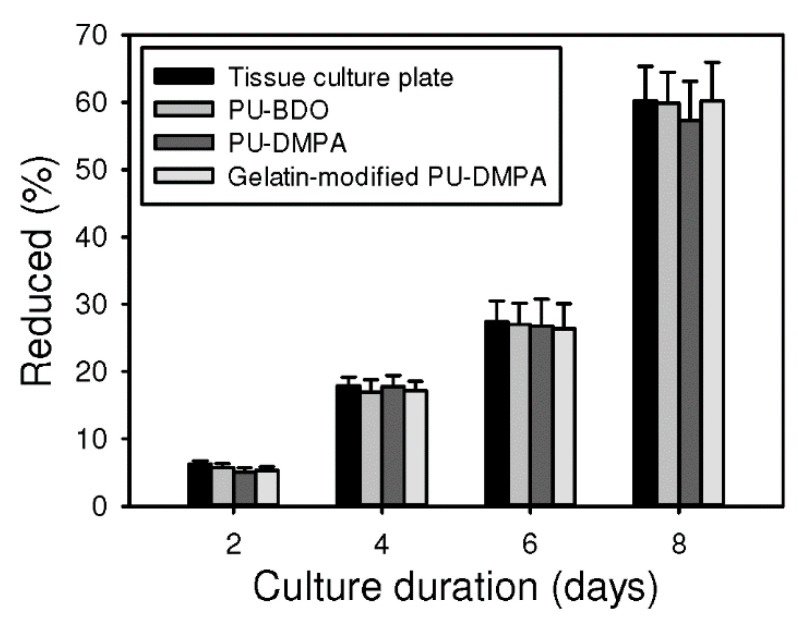
Growth of HUASMCs cultured alone in tissue culture plates, with the PU-BDO, PU-DMPA, and gelatin-modified PU-DMPA, respectively, in terms of the percentage of reduced Alamar blue.

After gelatin modification, the water contact angle of the PU-DMPA films slightly decreased to 57 ± 2.8°. The reduction in contact angle was not significant, however. Figure 6 shows that there were no differences in the metabolic activity of HUASMCs cultured with the PU-DMPA, gelatin-modified PU-DMPA and PU-BDO cast films and those grown alone in tissue culture plates throughout the eight-day culture, indicating their excellent cytocompatibility. 

**Figure 7:**
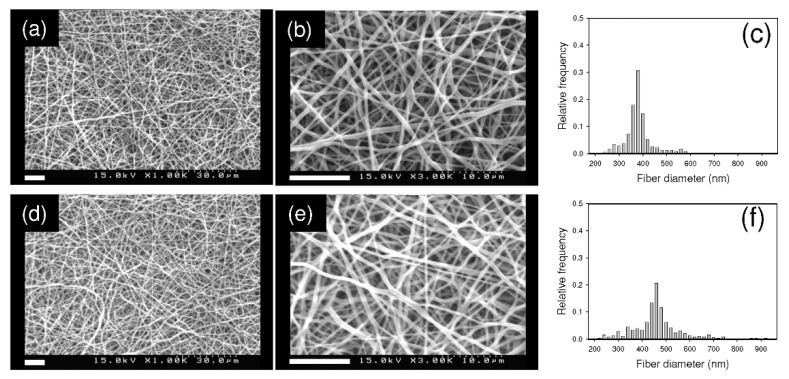
SEM images and diameter distributions of the as-spun PU-DMPA fibers (**a**–**c**) and the gelatin-modified PU-DMAP fibers (**d**–**f**); (**b**,**e**) are at higher magnifications. The scale bar at the bottom left of SEM images is 10 µm.

We attempt to use the PU-DMPA for fabricating tissue engineering scaffolds. As electrospinning has many advantages over conventional methods in this regard (e.g., fibers with the capability of contact guidance), efforts were made to fabricate electrospun PU-DMPA fibrous membranes. Despite the relatively low molecular weight of the PU-DMPA, bead free and drop free electrospun fibers were obtained when the concentration of the PU-DMPA solution was increased to 15% (*w/v*). The presence of carboxyl groups in the PU-DMPA was demonstrated by immobilization of gelatin on the as-spun PU-DMPA fibrous membrane using carbodiimide chemistry. The gelatin treatment significantly decreased the porosity of as-spun membranes from 78 ± 3% to 67 ± 7%. Figure 7 shows SEM images of the electrospun PU-DMPA fibrous membrane and the gelatin-modified counterparts. The diameter of gelatin-modified fibers (451 ± 144 nm) was significantly greater than that of as-spun fibers (380 ± 97 nm) (*p* < 0.05).

**Figure 8:**
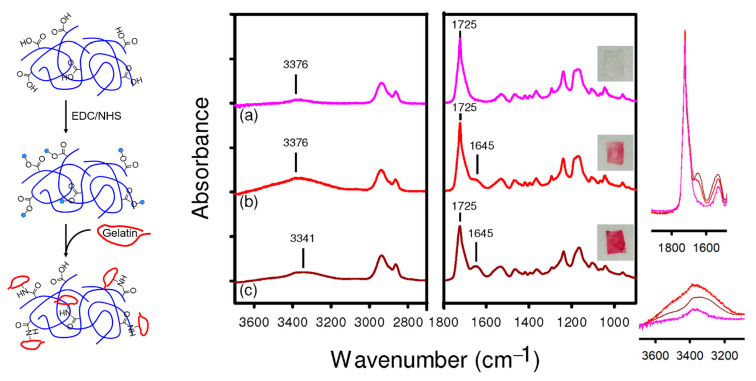
**Left**: schematic of the gelatin immobilization on the PU-DMPA electrospun fibers. **Right**: ATR-FTIR spectra of the PU-DMPA solid film (**a**), the gelatin-modified PU-DMPA solid film (**b**), and the gelatin-modified PU-DMPA electrospun fibrous mesh (**c**). The inserts on the right illustrate their corresponding Ponceau S staining.

Figure 8 shows the FT-IR spectra of the gelatin-modified PU-DMPA cast film, the gelatin-modified electrospun PU-DMPA membrane, and a negative control. The negative control was prepared by simply placing a nonactivated PU-DMPA cast film in gelatin solution followed by washing with deionized water. The characteristic peak of the gelatin amide I at 1645 cm^−1^ and the H-bonded O-H stretching broad peak extending from 3680 cm^−1^ to 3150 cm^−1^ indicate the successful immobilization of gelatin on the gelatin-modified specimens. There was no difference between the spectra of the negative control and the original PU-DMPA cast film. Ponceau S staining also confirmed the presence of gelatin on the gelatin-modified specimens.

**Figure 9:**
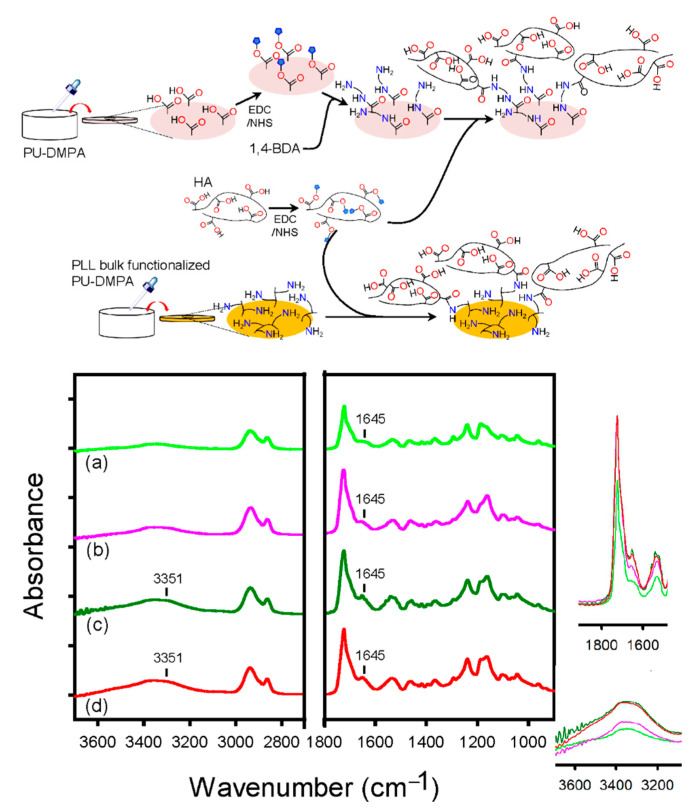
Top: schematic of the amino functionalization of the PU-DMPA cast film and bulk polymer solution by 1,4-BDA and PLL, respectively, followed by the incorporation of HA. Bottom: ATR-FTIR spectra of the BDA-modified PU-DMPA (**a**), the PLL-modified PU-DMPA (**b**), the HA-modified PU-DMPA-BDA (**c**), and the HA-modified PU-DMPA-PLL (**d**).

For more functionalization routes of the PU-DMPA, we endowed the PU-DMPA with amino groups using carbodiimide chemistry. The amino functionalization was performed on the PU-DMPA film surface or in a homogeneous solution of the PU-DMPA in THF. Figure 9 shows the FT-IR spectra of the PU-DMPA-BDA, the PU-DMPA-PLL, the PU-DMPA-BDA-HA, and the PU-DMPA-PLL-HA. The spectra of the PU-DMPA-BDA and the PU-DMPA-PLL showed the successful conjugation of amino groups either on the PU-DMPA film surface or in the PU-DMPA backbone. The presence of amino groups on the specimen surface was further confirmed by Ponceau S staining (data not shown). The spectra of the PU-DMPA-BDA-HA and the PU-DMPA-PLL-HA displayed a characteristic peak at 1645 cm^−1^, which is due to C=O stretching of an amide, and a broad peak centered about 3351 cm^−1^, which is attributed to profound H-bonded O-H stretching in HA, both indicating the successful grafting of HA. Upon the immobilization of HA, the contact angles of the PU-DMPA-BDA-HA (33.3 ± 4.3°) and the PU-DMPA-PLL-HA (28.6 ± 3.7°) cast films decreased significantly compared to that of the PU-DMPA-BDA (54.9 ± 4.0°) and PU-DMPA-PLL (54.0 ± 2.2°) films, respectively. 

**Figure 10:**
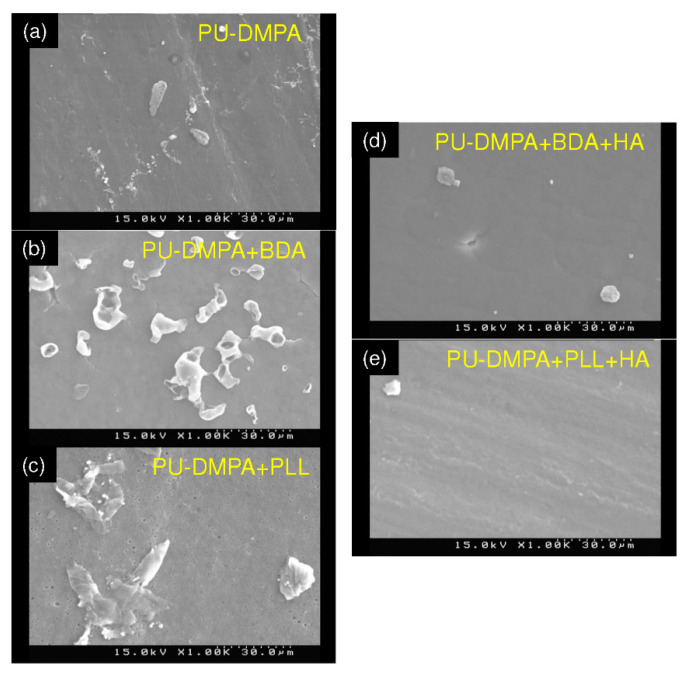
SEM images of platelets adhered onto the surface of the PU-DMPA (**a**), the BDA-modified PU-DMPA (**b**), the PLL-modified PU-DMPA (**c**), the HA-modified PU-DMPA-BDA (**d**), and the HA-modified PU-DMPA-PLL (**e**).

The HA-modified surface of the PU-DMPA-BDA and the PU-DMPA-PLL films were subjected to platelet adhesion tests with the PU-DMPA, PU-DMPA-BDA, and PU-DMPA-PLL films serving as controls. A material that has antithrombotic ability might be applied in the development of blood contact devices. Figure 10 shows the SEM images of the morphology of platelets adhered on the five surfaces. SEM showed less platelet adhesion on the surface of the PU-DMPA-BDA-HA and the PU-DMPA-PLL-HA films. The attached platelets, if any, had round shapes. The PU-DMPA-PLL-HA membrane even had less platelet adhesion than the PU-DMPA-BDA-HA. The results may be attributed to more amino groups in PLL and hence more HA conjugation on the PU-DMPA-PLL. Despite the limited number of carboxyl groups in the PU-DMPA backbone, the number of available amino groups was multiplied with the use of PLL as a linker. Obvious platelet adhesion on the PU-DMPA-BDA and the PU-DMPA-PLL was noted possibly due to the positive charge of amino groups. Interestingly, the PU-DMPA surface also displayed only few adhered platelets, perhaps due to the negative charged of the carboxyl groups. Hong et al. also found reduced platelet adhesion on the surface of their poly(ester urethane) bearing carboxyl groups [18]. This contradicts the finding in an earlier study; however, note that hydroxytelechelic polybutadiene was used for soft segments in said study [38]. 

Caution must be taken as surface modification allows imparting materials with specific functions, but the effect fades out as the material surface degrades. Incorporation of pendant reactive groups by bulk modification for subsequent functionalization would be more endurable. In this study, both approaches were performed to amino-functionalize the PU-DMPA. The immobilization of HA on the amino-functionalized PU-DMPA film was performed by surface modification, however.

There are a few limitations in this study. The number of carboxyl groups in the PU-DMPA was determined by the DMPA content. In this study, isocyanate-terminated PCL prepolymer was formed prior to the reaction with equimolar amount of DMPA, which resulted in an alternating copolymer—that is, the carboxyl groups were evenly distributed in the PU-DMPA backbone. We did not attempt to manipulate the DMPA content by mixing PCL diol with DMPA when forming the prepolymer nor by changing the ratio of PCL diol and DMPA chain extender, which could result in block copolymers with unpredictable structures. The changes in the DMPA content might increase the functionalizability of the polyurethane but could alter the mechanical properties of the polyurethane, which deserves a systematic investigation. It was suggested an increase in the DMPA content could reduce the molecular weight of the polymer [18], however. Note that polymer chain entanglement in solutions, which is closely associated with the molecular weight of a polymer, is an important prerequisite for electrospinning of the polymer. The carboxyl group in DMPA may react with isocyanates, although the steric hindrance that is due to the methyl group in DMPA could impede this reaction [39]. Using chain extender with a protecting group may reduce the consumption of carboxyl groups during synthesis [40]. The toxicity of tin-based catalysts remains a concern despite no obvious cytotoxicity being observed. Sn(Oct)_2_ may be replaced by amine catalysts such as triethylenediamine, which can be removed by water. Finally, due to the improved hydrophilicity of the PU-DMPA compared with the PU-BDO, the degradation rate of the PU-DMPA might be increased. The biodegradability of the PU-DMPA compared to the PU-BDO may be worth a thorough investigation. 

## 4. Conclusions

The PU-DMPA, based on a PCL soft segment, a HDI hard segment and a DMPA chain extender, was synthesized successfully as designed. The time required to complete the chain extension of the PU-DMPA was tremendously longer than that of the control PU-BDO (4 days vs. 24 h) and the PU-DMPA had a much lower molecular weight than the PU-BDO. The melting enthalpy of the PU-DMPA was greater than that of the PU-BDO. The PU-DMPA had a higher initial modulus and a lower linear modulus and ultimate strength than the PU-BDO, whereas the elongation at break of both the polyurethanes were comparable. The PU-DMPA was more hydrophilic than the PU-BDO. The different properties and behaviors might be attributed to the carboxyl groups in the PU-DMPA. Both the polyurethanes exhibited no cytotoxicity. The improved hydrophilicity of the PU-DMPA manifested better cell adhesion. Bead free and drop free electrospun PU-DMPA fibrous membranes with a narrow fiber diameter distribution were successfully fabricated using a relatively concentrated PU-DMPA solution.

Because of the presence of carboxyl groups in the PU-DMPA backbone, compounds containing amino groups can be conjugated to the PU-DMPA using carbodiimide chemistry. Compounds containing carboxyl groups, on the other hand, can be immobilized to the amino-functionalized PU-DMPA. Gelatin was successfully conjugated to the electrospun PU-DMPA membrane using carbodiimide chemistry. HA was immobilized on the amino-functionalized PU-DMPA cast film. Reduced platelet adhesion of the HA-modified PU-DMPA was noted. The PU-DMPA has the potential to be used as a scaffold material for soft tissue engineering.

## Data Availability

The data presented in this study are available on request from the corresponding author.

## Supplementary Materials

The following are available online at https://www.mdpi.com/article/10.3390/polym13091527/s1, Figure S1: GPC chromatograms of the PU-DMPA and the PU-BDO.
