# Supplementary figures and images for: Synthesis, Characterization, and Electrospinning of a Functionalizable, Polycaprolactone-Based Polyurethane for Soft Tissue Engineering
Source: Polymers (Basel). 2021 May 10;13(9):1527. doi: 10.3390/polym13091527 (PMC8126094; doi:10.3390/polym13091527)

## Slide 1
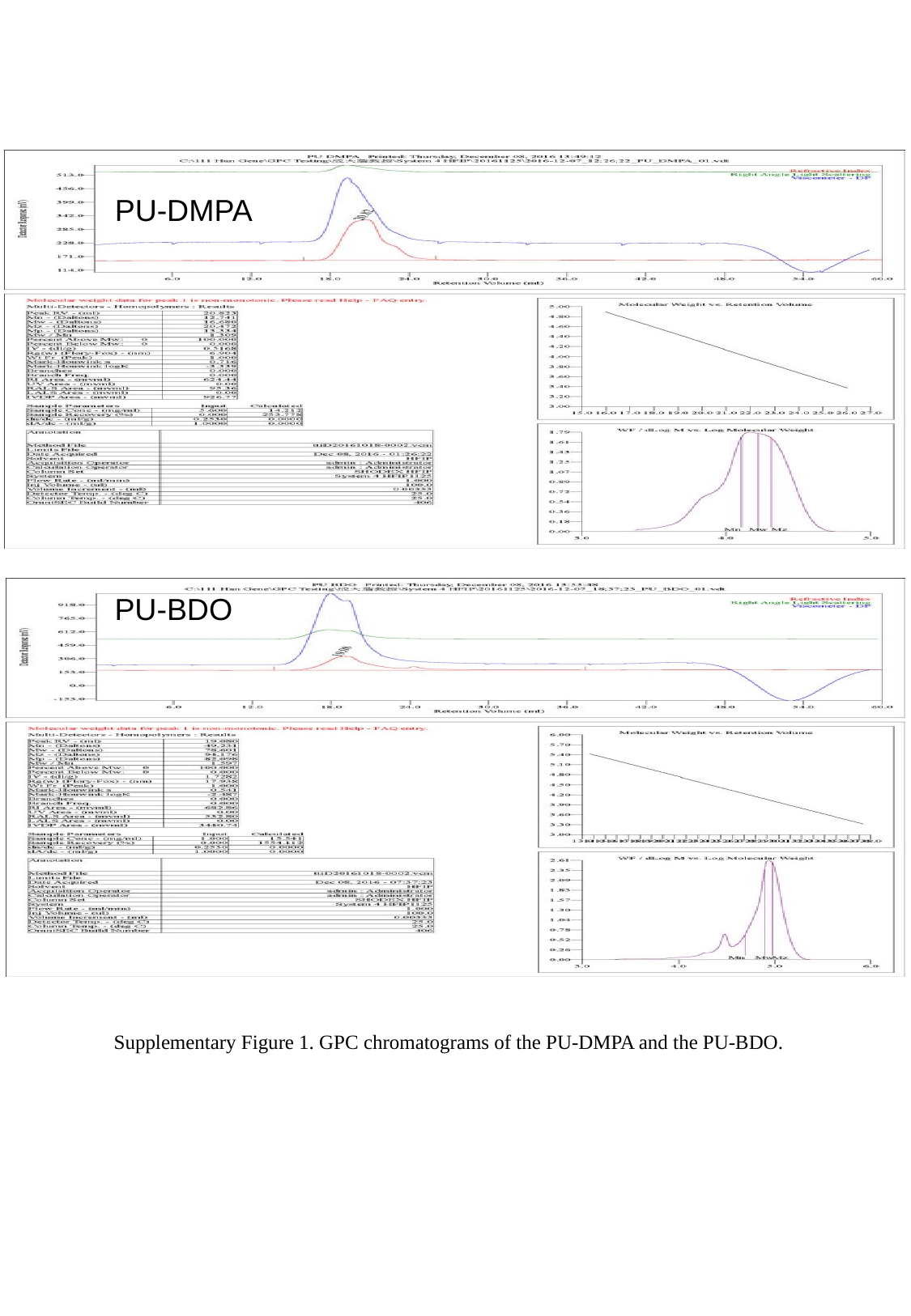

PU-DMPA
PU-BDO
Supplementary Figure 1. GPC chromatograms of the PU-DMPA and the PU-BDO.

Supplement: Supplementary file 1 [file polymers-13-01527-s001.zip › polymers-1195500-supplementary.pptx]
